# Extinction Coefficient Modulation of MoO_3_ Films Doped with Plasmonic Nanoparticles: From an Effective Medium Theory Description

**DOI:** 10.3390/nano11082050

**Published:** 2021-08-12

**Authors:** Gesuri Morales-Luna, Michael Morales-Luna

**Affiliations:** 1 Departamento de Física y Matemáticas, Universidad Iberoamericana Ciudad de Mexico, Prolongación Paseo de la Reforma 880, Ciudad de Mexico 01219, Mexico; 2 Escuela de Arquitectura y Ciencias del Hábitat, Universidad de Monterrey, Av. Ignacio, Morones Prieto 4500 Pte., San Pedro Garza García 66238, Mexico

**Keywords:** extinction coefficient, effective medium theory, solar cells

## Abstract

This work focused on the application of the effective medium theory to describe the extinction coefficient (*Q_ext_*) in molybdenum trioxide (MoO_3_) doped with different kinds of plasmonic nanoparticles, such as silver (Ag), gold (Au), and copper (Cu). Usually, in studies of these materials, it is normal to analyze the transmission or absorption spectra. However, the effect of this type or size of nanoparticles on the spectra is not as remarkable as the effect that is found by analyzing the *Q_ext_* of MoO_3_. It was shown that the *β*-phase of MoO_3_ enhanced the intensity response of the *Q_ext_* when compared to the *α*-phase of MoO_3_. With a nanoparticle size of 5 nm, the Ag-doped MoO_3_ was the configuration that presents the best response in *Q_ext_*. On the other hand, Cu nanoparticles with a radius of 20 nm embedded in MoO_3_ was the configuration that presented intensities in *Q_ext_* similar to the cases of Au and Ag nanoparticles. Therefore, implementing the effective medium theory can serve as a guide for experimental researchers for the application of these materials as an absorbing layer in photovoltaic cells.

## 1. Introduction

Energy production in the world has become a problem of great interest in different research groups. It has been found that anthropogenic activities are the cause of constantly increasing greenhouse gas emissions. This is due to the strong dependence that the current technology has on the consumption of fossil fuels. Being that 85% of the energy production depends on the burning of these non-renewable resources. However, the alternative technologies that are growing in energy production (~15%) are those that depend on the use of renewable resources such as wind or solar radiation, to mention the most developed alternatives [1]. Particularly, the use of solar radiation has attracted the greatest interest in the theoretical area, as well as in the design and development of solar devices like photovoltaic systems, or solar collectors [2]. In solar cells applications, there are a large number of solar cell types such as silicon, amorphous silicon, chalcogenides, and perovskites, to name just a few [3,4,5,6]. These types of solar cells have been studied as single-junction and multiple-junction or tandem [7]. Principally, for a multiple-junction, these devices consist of different layers that have different aims but together can increase the efficiency of the module [7,8,9].

This work will focus on the study of the absorbing layer, which in this case will be doped by metallic nanoparticles. Much of the improvement processes in the absorbing film consists in the light scattering or absorbing by resonant nanoparticles [10]. A certain amount of light can be trapped, and an electron–hole pair can be generated, but the amount of light that cannot be absorbed can be reflected by a metal back layer in the solar cell arrangement and eventually, the light can interact again with the metallic nanoparticles, enhancing the generation of electron–hole pairs [11]. To enhance the optical properties, materials with a greatest potential to be applied as an absorbing layer are the transition metal oxides (TMO’s) and among the most studied are molybdenum oxides (MoO*_x_*) [12]. Depending on the desired applications, MoO*_x_* can be deposited or synthesized by physical or chemical techniques, respectively [13]. The deposition or synthesis techniques allow us to modify the characteristics of the oxide in order to enhance the desired properties according to the application [14]. In addition, different configurations of deposition have been developed, such as MoO_3_/CdSe or MoO_3_/CdS or Au/MoO_3_ [15,16,17]. The purpose of studying these configurations is to enhanced the optical absorbance of molybdenum trioxide (MoO_3_). Also, the MoO_3_ thin films, has been doped with zinc selenide (ZnSe), copper (Cu), silver (Ag) or gold (Au) [18,19,20,21].

This latter configuration has generated significant interest due to the ability to modulate the absorbance in the visible range, a feature of great importance in photocatalytic applications, biological sensing, smart windows, and photovoltaic cells [22]. This property is attributed to a phenomenon called superficial plasmon resonance (SPR) [23,24]. In the study of photovoltaic cells, there are many phenomena that are studied with the aim of enhancing the plasmonic harvesting of sunlight in solar cells. To mention some of them, hot-electrons injected from plasmonic nanoparticles [25], plasmon-induced absorption [26], plasmon-induced resonance energy transfer (PIRET) [27], plasmon-mediated processes [28]. Recently, our group has developed a theoretical study that describes in detail the behavior of the SPR signal intensity in the MoO_3_ transmittance spectrum when it varying the concentration of Au nanoparticles, as well as the phase of MoO_3_ [22]. However, in that work, the effect of the nanoparticle size in the changes in transmittance spectrum; particularly in the SPR signal, did not show a strong dependency.

In the study of solar cells, the absorption spectra of the oxides are often analyzed to determine the optical properties [29,30,31,32,33,34]. An alternative to studying the absorbance spectra and that few research groups have paid attention to, is the extinction coefficient analysis of these materials [35,36,37]. Consequently, this work will focus on the analysis of the effects that different nanoparticle sizes produce on the extinction coefficients of MoO_3_ thin films. The analysis presented in this work uses the effective medium theory proposed by van de Hulst [38], which is commonly named the effective refractive index (RI) of van de Hulst [39,40]. It is important to highlight at this point that, as far as we know, the RI model has not been used to compute the absorbance or the extinction coefficient of this type of thin films. The extinction coefficient analysis allows us to study the changes of the optical response of this type of film in a more detailed way, avoiding the inherent errors and experimental variability in the deposition or syntheses of this type of thin film. The extinction coefficient provides information due to absorption and/or scattering, which provides more information than the absorption coefficient would give. Although they are physical quantities related to each other, the effects that nanoparticles provide due to the imaginary part of the system show a magnification in the extinction spectrum compared to the absorption spectrum, thus making these effects easier to read due to the particles embedded in the film. Hence, the effective medium theory (EMT) needs to take into account some important conditions, the most important condition of the EMT proposed by van de Hulst being that the mutual distances of particles should be much bigger than the wavelength. Additionally, the forward scattering is considered, the value of which is decisive for the extinction and is the parameter that this work is focused on, which is why EMT was chosen. Thus, the forward scattering is one of the main differences between van de Hulst’s and other effective medium theories, like Maxwell–Garnett [41], which is common to apply this model for granular topology. The EMT proposed by van de Hulst is used in this work to describe MoO_3_ thin films doped by different resonant nanoparticles (NPs) such as silver (Ag), gold (Au), and copper (Cu). The parameters that can be changed to modify the optical properties of the doped thin films are the thickness of the film, the volume filling fraction (*f*), and the nanoparticle size (*a*). Finally, it will be shown that the modification of extinction coefficient depends strongly on these last two parameters. EMT is one of the most important theories for computing the optical properties of doped thin films combined with the transmittance and reflectance coefficients using the well-known Fresnel coefficients [42,43].

## 2. Theoretical Model

**Figure 1:**
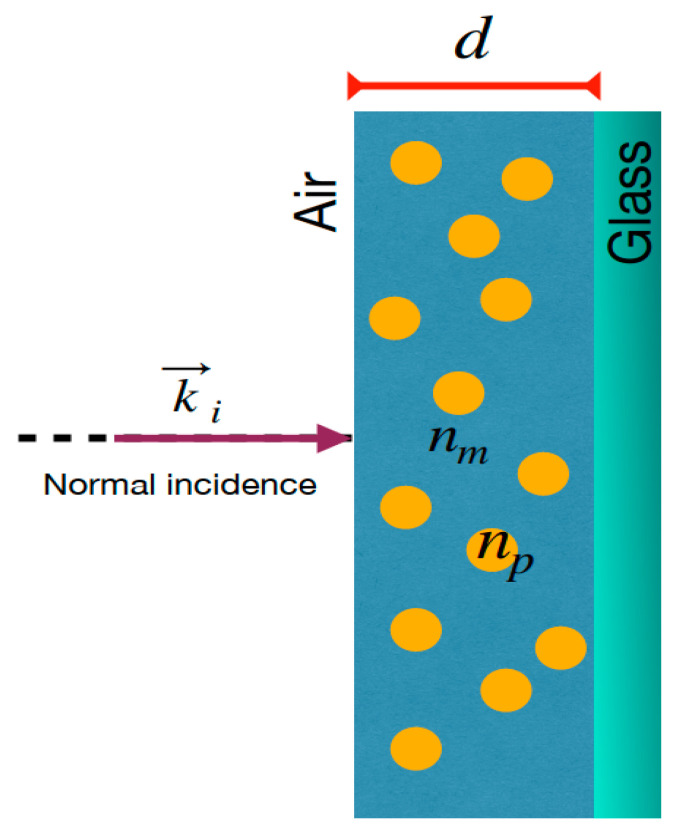
Schematic representation of MoO_3_ thin film doped by metallic nanoparticles on a glass substrate showing the normal incidence of the light. ${\vec{k}}_{i}$ is the incident wave vector, $n_{m}$ and $n_{p}$ are the refractive index of the matrix medium and the refractive index of the nanoparticles, respectively. d is the thickness of the doped thin film.

To determine the absorbance and the extinction coefficient (*Q_ext_*), it is necessary to consider the Fresnel coefficients. These coefficients are defined by

(1) $$r_{ij}=\frac{k_{iz}-k_{jz}}{k_{iz}+k_{jz}}$$

for the transverse electric, TE, polarization and

(2) $$r_{ij}=\frac{n_{j}^{2}k_{iz}-n_{i}^{2}k_{jz}}{n_{j}^{2}k_{iz}+n_{i}^{2}k_{jz}}$$

for the transverse magnetic, TM, polarization. The subscript *i*, *j* depends on the interface that is analyzed (see Figure 1). The $k_{lz}$, where l=i, j is the *z*-component of the wavenumber and takes the form of $k_{lz}=k_{o}\sqrt{n_{l}^{2}-n_{1}^{2}\sin^{2}\theta_{1}}$, where $k_{o}=2\pi /\lambda$ is the wave number in a vacuum and λ is the wavelength. To calculate the reflectance of the entire system, the composed formula of reflectance [42] is needed which is defined as,

(3) $$r_{123}=\frac{r_{12}+r_{23}\exp\left[2ik_{z}^{eff}d\right]}{1+r_{12}r_{23}\exp\left[2ik_{z}^{eff}d\right]}$$

where *d* is the thickness of the thin film. To simulate the transmittance, the next expression is considered,

(4) $$t_{123}=\frac{t_{12}t_{23}\exp\left[ik_{z}^{eff}d\right]}{1+t_{12}t_{23}\exp\left[2ik_{z}^{eff}d\right]}$$

where $k_{z}^{eff}$ is the wavenumber in the effective medium, defined as

(5) $$k_{z}^{eff}=k_{o}\sqrt{n_{2}^{2}-n_{1}^{2}\sin^{2}\theta_{i}+2n_{2}^{2}\left[\frac{3if}{2{\left(k_{o}n_{2}a\right)}^{3}}S\left(0\right)\right]}$$

where $n_{l}$ is the refractive index in each medium, $\theta_{i}$ is the angle of incidence, *a* is the radius of the particle, f is the volume filling fraction, and S(0) is the forward-scattering amplitude of the NPs inside the matrix [44]. For the transmission coefficients, the following equations were used:

(6) $$t_{ij}=\frac{2k_{iz}}{k_{iz}+k_{jz}}$$

for the TE polarization, and

(7) $$t_{ij}=\frac{2n_{j}n_{i}k_{iz}}{n_{i}^{2}k_{jz}+n_{j}^{2}k_{iz}}$$

for the TM polarization. Finally, it is necessary to consider the reflectance and the transmittance which are defined as

(8) $$R={\left|r_{123}\right|}^{2}$$

and

(9) T=Re[k3zk1z]|t123|2

It is necessary to remember that this work will describe thin films doped by resonant NPs, and the effective medium model that will be used to describe this, will be the van de Hulst effective RI [38], which is given by

(10) $$n_{vdH}=n_{m}\left[1+\frac{3if}{2x^{3}}S\left(0\right)\right]$$

where $n_{m}$ is the refractive index of the medium where the NPs are immersed, which in our case is the molybdenum trioxide in alpha and beta phase [45], $x=k_{o}n_{m}a$ is the parameter size, where a is the radius of the NPs and the other parameters have already been defined.

As already mentioned, this paper is focused principally on the description of the extinction coefficient. First of all, the absorption coefficient is defined as

(11) $$\alpha =\frac{1}{d}\ln\frac{{\left(1-R\right)}^{2}}{T}$$

where d is the thickness of the thin film, and R and T are the optical reflectance and transmittance defined in Equations (8) and (9). Finally, in thin films, the extinction coefficient is defined as

(12) $$Q_{ext}=\frac{\alpha \lambda }{4\pi }$$

which depends on the absorption, α, and the wavelength, λ [46].

## 3. Absorbance and Extinction Coefficients of MoO_3_ Thin Films Doped by Resonant NPs

In order to analyze the effect of the resonant NPs embedded in a MoO_3_ thin film (at alpha and beta phases), the absorbance and extinction coefficient will be shown in this section, so that different cases of doping NPs can be presented.

### 3.1. α-Phase of MoO_3_ Doped with Ag, Au, and Cu-NPs

**Figure 2:**
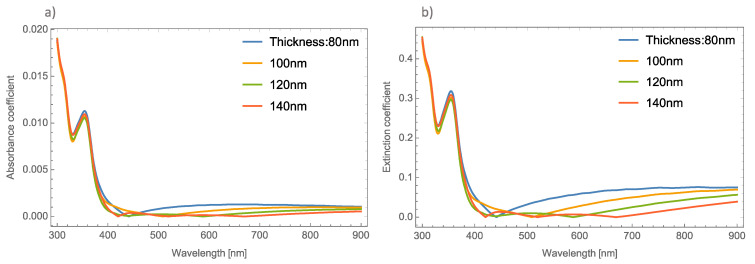
(**a**) Absorbance coefficient for different thicknesses of the MoO_3_ thin film without resonant NPs. (**b**) Extinction coefficient for different thicknesses of the un-doped MoO_3_ thin film.

Figure 2a,b, depicts the absorbance and the extinction coefficients; respectively, at different thicknesses of the un-doped MoO_3_ thin film at the *α*-phase.

As Figure 2 shows, there is no great difference in the absorbance spectra at different thicknesses of the MoO_3_ thin films. However, greater changes can be seen in the extinction coefficient. One thing that is important to highlight in the absorbance spectra is that it shows a shift exactly in the absorbance zero value. This absorbance zero value means that at this wavelength the reflected light is small compared with the transmittance. It is essential to emphasize this behavior, because if the medium was switched to a non-absorbing medium, this behavior could be modified [47]. Remember that the effective refractive index proposed by van de Hulst considers the whole contribution of the matrix medium (MoO_3_). For this oxide, the refractive index has a real and an imaginary part, which indicates that the main contribution of this phenomenon is the thin film of the MoO_3_. This can be seen in Figure 2, where the cases of un-doped MoO_3_ films are presented. The figure shows the evolution of the extinction coefficient zero value as the thickness of the film increases. It is well known that the plasmonic nanoparticles (in this case silver, gold and copper) have real and imaginary refractive indices that are also considered in the computed transmission and reflection coefficients of the system but the contribution of the metallic nanoparticles at these wavelengths can be negligible and we can conclude that this phenomenon occurs due to the molybdenum trioxide. In our case, all the calculations considered a MoO_3_ matrix, which is an absorbing medium. As can be seen in Figure 2a, the absorbance zero value shifts to higher wavelengths as the film thickness increases, for example, in the case of 80 nm, the absorbance zero value is at 440 nm, when the thickness is 100 nm, this value is 510 nm. It is necessary to point out that for the case of a film with a thickness of 140 nm, there are three absorbance zero values. In Figure 2a this tendency is not so clear, but in Figure 2b the phenomenon can be clearly seen, with extinction or absorption equal to zero at wavelengths of 420 nm, 520 nm, and 670 nm. On the other hand, Figure 2b depicts the evolution of the *Q_ext_* where it can be seen that there is a bigger shift in where the absorbance becomes zero.

**Figure 3:**
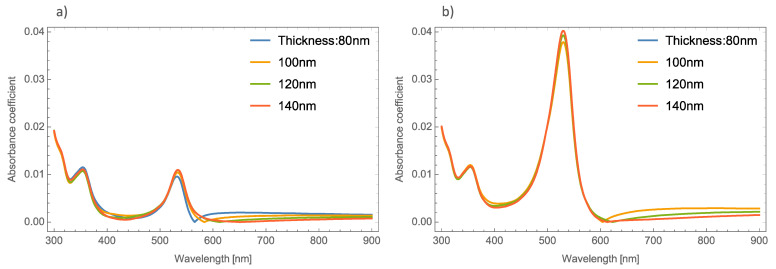
Absorbance coefficient of the MoO_3_ thin film doped with Ag-NPs of radius of 5 nm, (**a**) volume filling fraction of Ag-NPs is 1% and (**b**) the volume filling fraction of Ag-NPs is 5%. The different thicknesses are labeled.

Now we examine what happens to the absorbance spectrum in a simulated orthorhombic MoO_3_ thin film with different thicknesses and doped with Ag–nanoparticles with different values of radii and volume filling fractions. Figure 3a,b depicts the absorbance coefficient of MoO_3_ thin films at different thicknesses at two different volume filling fractions, one and five percent, respectively. As can be seen in Figure 3a, the absorbance coefficient shows a peak centered at 520 nm which is characteristic of the resonant NPs, and additionally, the same peculiar behavior (redshift) in the absorbance zero value is observed as mentioned above. For example, for the 80 nm thickness, the absorbance zero value was found at 565 nm, for 10 nm of thickness this value shifts to 585 nm and for the other two cases, the absorbance zero value is not well defined. Furthermore, the SPR signal is more intense as the volume filling fraction increases, which was expected to obtain due to previous studies, see Figure 3b. Now the absorbance zero value redshift is not so marked as in the lowest volume filling fraction. As can be seen in Figure 3, the SPR signal does not suffer as much modification as the thickness of the MoO_3_ film increases. Due to this behavior the thickness was set at 120 nm for the following simulations.

**Figure 4:**
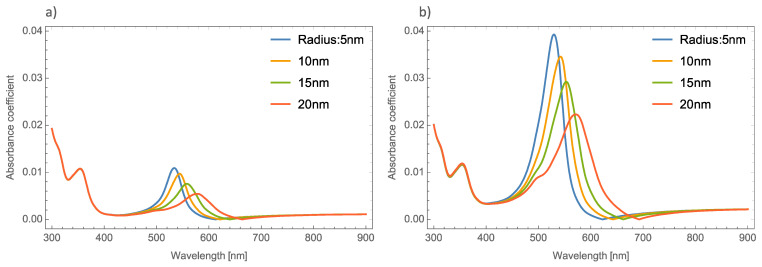
Absorbance coefficient of the MoO_3_ thin film doped with Ag-NPs with a thickness value of 120 nm, (**a**) volume filling fraction of 1% and (**b**) volume filling fraction of 5%. The different NPs radii are indicated.

Figure 4a,b displays the absorbance coefficient of Ag NPs embedded in the MoO_3_ thin films for two different filling fractions, one and five percent, respectively. As can be seen in this figure, the variation of the nanoparticle radius significantly modifies the resonant signal; as the nanoparticle size increases, the intensity of the resonant signal decreases. The absorbance zero values in this Figure are still redshifted. In Figure 4b when the nanoparticle size increases and the volume filling fraction increases, the resonant signal increases, as was mentioned above, and the maximum of the peak undergoes a redshift around 65 nm in this case, the absorbance zero value still displays a redshift.

**Figure 5:**
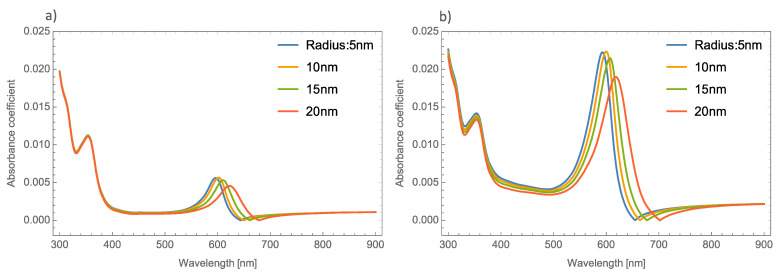
Absorbance coefficient of the MoO_3_ thin film doped with Au-NPs with a thickness of 120 nm, (**a**) volume filling fraction of 1% and (**b**) volume filling fraction of 5%. The different NPs radii are indicated.

The absorbance spectra of Au and Cu NPs show a similar behavior in both phases of MoO_3_ (alpha and beta) as the absorbance spectra of silver NPs. For simplicity, only the Au nanoparticle absorbance spectra was shown. Therefore, we can begin to analyze the absorbance coefficient using different radii of Au NPs. Figure 5a,b shows the absorbance coefficient of MoO_3_ thin films doped with Au NPs at one and five percent of the volume filling fraction, respectively. As was mentioned, Figure 5a shows similar behavior to that presented by the Ag nanoparticles embedded in the MoO_3_ matrix. The obtained redshift of SPR peaks is 25 nm, and the obtained shift for the absorbance zero value is 36 nm. A peculiar aspect that can see in this figure is the highest intensity of the SPR peak for a radius of 10 nm of the NPs, after this value the intensity of the SPR peaks decreases. Figure 5b displays the evolution of the absorbance as the radius increases at 5% of Au NPs, here the maximum intensity of absorbance peaks is for a radius of 10 nm. It is important to note at this point that the behavior of the absorbance spectra with the changing radii of NPs is similar to the case of MoO_3_ films doped with Ag NPs. Analyzing this figure, the SPR signal displays a redshift of 27 nm and the obtained redshift for the absorbance coefficient zero value is 47 nm. When Figure 5 is compared with the case of silver nanoparticles (Figure 4), the maximum value of the resonance peak is redshifted by 40 nm, which is lower than the Ag NPs case. For the absorbance zero value, the redshift is 23 nm less than the case of molybdenum trioxide doped with silver nanoparticles.

As already mentioned, there is a dependence of the radius of the NPs on the absorbance coefficient principally in the intensity of the SPR signal. A difference of 15 nm in NP radius, shifts the resonant maximum peak around 60 nm. In this case, the thickness was fixed at 120 nm and again a redshift can be seen in the absorbance zero value from 620 to 660 nm. As was discussed, this behavior is due to the presence of the NPs that affect the shifts of the maximum absorbance peaks as the volume filling fraction increases. These characteristics in the absorption spectra indicates the need to study in a more precise way the interaction between the light and the MoO_3_ thin films with the extinction coefficient as an alternative to the absorbance studies. Therefore, as already mentioned, analyzing the *Q_ext_* could be a novel alternative to study the behavior of the NPs embedded in the MoO_3_ matrix.

**Figure 6:**
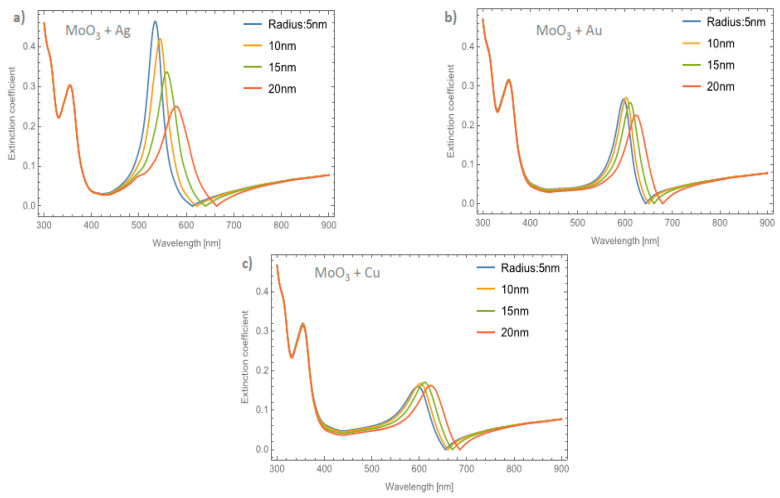
The extinction coefficient of the MoO_3_ thin film doped with (**a**) Ag-NPs, (**b**) Au-NPs, and (**c**) Cu-NPs for a volume filling fraction of 1%. In all spectra, the thickness was fixed to a value of 120 nm, and different NPs radii are labeled.

Figure 6a–c shows the behavior of the extinction coefficient of the MoO_3_ thin film doped with Ag, Au, and Cu NPs, respectively. The thickness was fixed to 120 nm and different radii of nanoparticles are displayed. The behavior of *Q_ext_* is similar to the absorbance coefficient but the peak intensity in this coefficient is more intense than the absorbance. If Figure 6a, Ag NPs doped MoO_3_, is compared with Figure 4a, it can be seen that analyzing the extinction coefficient could be more useful for a better description of the optical properties because the effects on the spectra are amplified. From Figure 6a, the *Q_ext_* zero values present a redshift of around 50 nm from the smallest radius (5 nm) to the highest radius (20 nm) additionally the maximum of the SPR peak shows a redshift of 45 nm. Figure 6b corresponds to the Au nanoparticles doped onto the MoO_3_ film, this figure displays a redshift of the extinction coefficient zero value of 36 nm and the obtained redshift for the maximum of the SPR, Δ_SPR-*α*_, peak is 27 nm. The Δ_SPR-*α*_ value was calculated by the difference in the maximum peak value for the case of the 5nm radius nanoparticles and the maximum peak value for the 20 nm radius nanoparticles. Comparing the redshift of the Au NPs at a 1% filling fraction with the Ag NPs; using the *Q_ext_*, the SPR redshift for the Au NPs is around 36 nm, while in the case of silver it is around 45 nm. Therefore, depending on the application it could be useful to change both resonances. In addition to the redshift of the resonant signal, there is also a change of intensity of the resonant signal, showing the importance of analyzing the extinction coefficient. Figure 6c displays the evolution of *Q_ext_* as the radii of the nanoparticles changes for the Cu nanoparticles embedded in MoO_3_ films. The obtained redshift value of the SPR peak is 28 nm and the obtained shift value of the zero of extinction coefficient is 31 nm. On the other hand, the intensity increases as the radius increases up to a radius of 15 nm where the highest intensity SPR peak occurs, after this value the SPR signal decreases.

**Figure 7:**
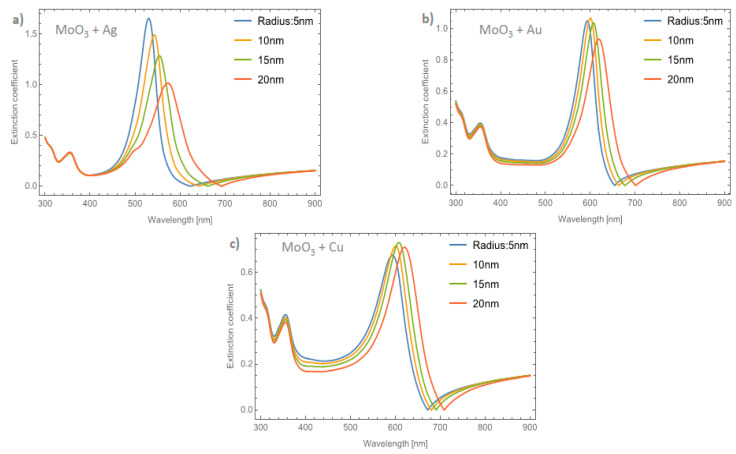
The extinction coefficient of the MoO_3_ thin film doped with (**a**) Ag-NPs, (**b**) Au-NPs, and (**c**) Cu-NPs for a volume filling fraction of 5%. In all spectra, the thickness was fixed to a value of 120 nm, and different NPs radii are labeled.

Figure 7a–c depicts the evolution of *Q_ext_* at a fixed volume filling fraction of 5% for the Ag, Au, and Cu NPs, respectively. If these plots are compared with Figure 4b it can be seen that the intensities of the peaks are higher. Analyzing the extinction coefficient zero values for the MoO_3_ film doped with Ag nanoparticles, see Figure 7a, it is observed that the redshift is around 70 nm and the shift in the maximum of the SPR peaks is around 43 nm. As has been shown, the resonant NPs have an important effect on the optical properties of the MoO_3_ thin film. Figure 7b depicts the evolution of *Q_ext_* as the radius of the Au-NPs is increased. The highest value of *Q_ext_* is for NPs with a radius of 10 nm. The obtained redshifts of the maximum of the SPR peak and for the extinction coefficient zero value are 26 and 47 nm, respectively. Figure 7c shows the *Q_ext_* evolution for a volume filling fraction of 5% of copper nanoparticles with different nanoparticles radius. As was observed in the other cases, the intensity of the SPR signals are greater than the ones shown in Figure 6c. It is evident that the MoO_3_ film doped with 5% of Cu NPs with a radius of 15 nm, is the sample that shows the most intense SPR signal. This behavior cannot be observed for gold and silver nanoparticles, so from these results, it can be seen that the nanoparticle radius correlates with the maximum SPR signal intensity and additionally with the type of nanoparticle embedded in MoO_3_ film. Hence, in the *α*-phase of MoO_3_, the intensity of the SPR signal in the extinction coefficient was modified by the presence of different radii of nanoparticles. In the case of Ag NPs doped MoO_3_ thin films, the signal with the highest value of the SPR signal is for a sample doped with nanoparticles with a radius of 5 nm. For the Au NPs doped MoO_3_ thin film the highest intensity of the SPR signal is for the doped thin film with nanoparticles with a radius of 10 nm. Finally, for Cu NPs doped MoO_3_ thin film the best response in the SPR signal is for nanoparticles with a radius of 15 nm.

Now we examine what happens with the extinction coefficient if the phase of the MoO_3_ thin film is changed using the same NP types as the *α*-phase. For simplicity, only the extinction coefficients will be presented because the spectra in shape are very similar to those presented in Figure 4. Here, are going to be presented the cases for the *β*-phase of MoO_3_.

### 3.2. β-Phase of MoO_3_ Doped with Ag, Au and Cu-NPs

**Figure 8:**
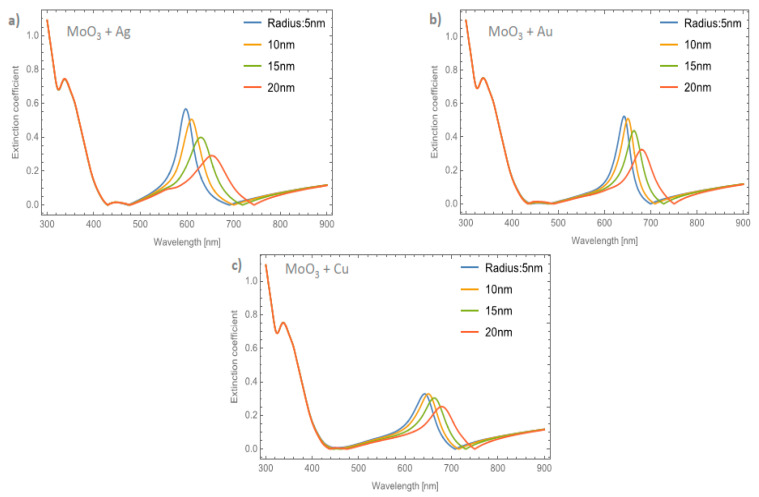
The extinction coefficient of the MoO_3_ thin film doped with (**a**) Ag-NPs, (**b**) Au-NPs, and (**c**) Cu-NPs for a volume filling fraction of 1%. In all spectra, the thickness was fixed to a value of 120 nm, and different NPs radii are labeled.

Figure 8a–c depicts the evolution of the *Q_ext_* of the *β*-MoO_3_ thin film (for a *f* = 1%) as the embedded nanoparticles change. The *β*-phase displays an amorphous structure; this feature is different from the *α*-phase which is displays a crystal structure. From the Figure, we can observe significant differences with respect to the α-MoO_3_ phase in two important points. The first one (see Figure 8a) is the redshift of the maximum resonance peak between the nanoparticles radii of 5 and 20 nm (Δ_SPR-_*_β_*). As can been seen, just changing the phase of the MoO_3_ the maximum resonance value was shifted by around 56 nm. Additionally, the extinction coefficient zero value undergoes a shift around 55 nm. The second point, is the width of the peak. To quantify this feature, the full width at half maximum (FWHM) was calculated. The obtained values range from 43.19 nm for a nanoparticle radius of 5 nm to 88.96 nm for a nanoparticle radius of 20 nm, the difference between these values being 45.77 nm (Δ_FWHM-_*_β_*). MoO_3_ doped with Au NPs, Figure 8b, displays a similar demeanor in the shift of the resonance peak to that presented in the silver nanoparticles cases. The Δ_SPR-*β*_ is 39 nm, which is bigger than the case of the *α*-phase at 27 nm. The value of the Δ_FWHM-*β*_ shift is 23.71 nm. Finally, the extinction coefficient zero value from this Figure changes by 51 nm. As we have already mentioned, the maximum resonance peak for the copper nanoparticles, undergoes a redshift from 642 nm to 679 nm (Δ_SPR-*β*_ = 37 nm) for a nanoparticle radius of 5 and 20 nm, respectively for the case of 1 % of volume filling fraction, see Figure 8c. The Δ_FWHM-*β*_ value was 17.08 nm which is 11.89 nm greater than the presented changes in the *α*-phase. The obtained zero value change of *Q_ext_* in this case is 42 nm.

**Figure 9:**
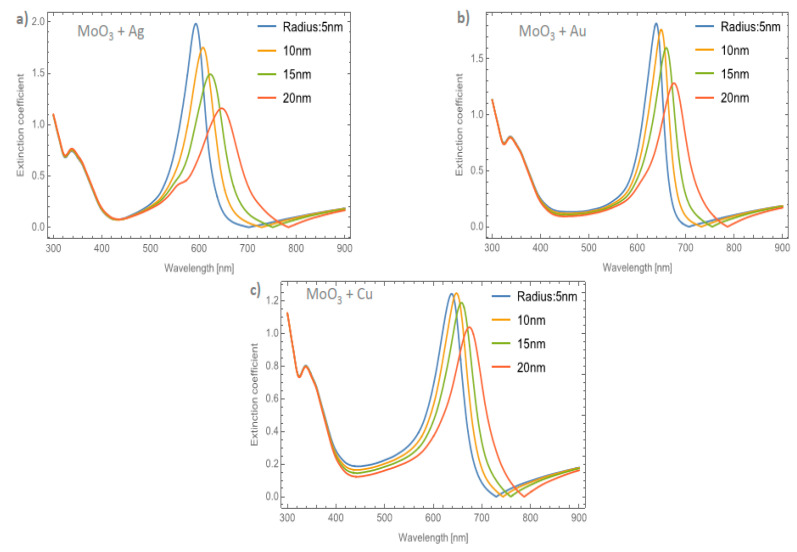
The extinction coefficient of the MoO_3_ thin film doped with (**a**) Ag-NPs, (**b**) Au-NPs, and (**c**) Cu-NPs for a volume filling fraction of 5%. In all spectra, the thickness was fixed to a value of 120 nm, and different NPs radii are labeled.

Figure 9a–c displays the evolution of the *Q_ext_* of the *β*-MoO_3_ thin film as the embedded nanoparticles change at a volume filling fraction of 5%, for Ag, Au, and Cu respectively. From Figure 9a for Ag doped MoO_3_, the Δ_SPR-*β*_ value is 54 nm. Additionally, the redshift value of the extinction coefficient zero value is 83 nm and the obtained Δ_FWHM-*β*_ value for this case is 40.63 nm. As can been seen, the redshift value on the maximum resonant peak and the FWHM values of the peaks are strongly affected by the type and features of the nanoparticle. For the MoO_3_ doped with Au nanoparticles, see Figure 9b, the Δ_SPR-*β*_ and Δ_FWHM-*β*_ values are 37 and 24.01 nm, respectively. The change in the extinction coefficient zero values for this case is 81 nm. Figure 9c displays the evolution of the *Q_ext_* as the nanoparticle radius changes at 5% of Cu NPs. The obtained values are Δ_SPR-*β*_ = 36 nm, the *Q_ext_* zero value shows a shift of 58 nm, and finally the Δ_FWHM-*β*_ = 17.72 nm. Notably, the behavior of the copper NPs that was presented in the *α*-phase is not present in this case. For the *α*-phase analysis there is an increase in the SPR signal intensity with the maximum depending on the doping nanoparticles used and the radius of the nanoparticles. In the *β*-phase, the maximum of the SPR signal of the extinction coefficient partially decreases as the radius of the NPs increases. As can been observed, there is a maximum for the *Q_ext_* for nanoparticles of 10 nm radius. One of the most important things, in this case, is that the resonance peaks are similar to the cases of the gold nanoparticles embedded in the MoO_3_ films but the widths of the spectra are greater than the gold NPs case. For the silver and gold NPs, the resonance peaks are narrower than the resonance peaks associated with the copper NPs. This demeanor in the extinction coefficient makes the copper NPs a good candidate for solar radiation absorber layer applications as in solar cells or solar condensers, instead of using silver or gold nanoparticles as dopants which are more expensive than copper nanoparticles. So, the range of the absorbance response of the MoO_3_ thin films doped with Cu NPs could be greater than the Au or Ag doped MoO_3_ thin films due to the wider extinction coefficient peaks covering more wavelengths of the spectrum.

As a summary, Table 1 is presented, where the resonant peak redshift values of the MoO_3_ thin films doped with different types and characteristics of NPs, as labeled in the table, are reported. A demeanor that is easy to observe from this table is that the Ag nanoparticles present the highest Δ_SPR_ values compared to the Au and Cu nanoparticles. On the other hand, the MoO_3_ thin films doped with Cu nanoparticles display the lowest changes in the Δ_SPR_ values in both phases of MoO_3_. As a complement to the discussion and to Table 1, Table 2 is reported, where the change in the FWHM values can be seen for the different doping configurations of the MoO_3_ thin film. From this table, it can be seen that the greatest broadening of the SPR signal occurs for Ag nanoparticles, as the radius of the nanoparticles increases and when the MoO_3_ is in its beta phase. In the same way as was observed for the displacement of the maximum resonant peak, the FWHM values that demonstrated lower changes are the cases of MoO_3_ thin films doped with Cu NPs.

**Figure 10:**
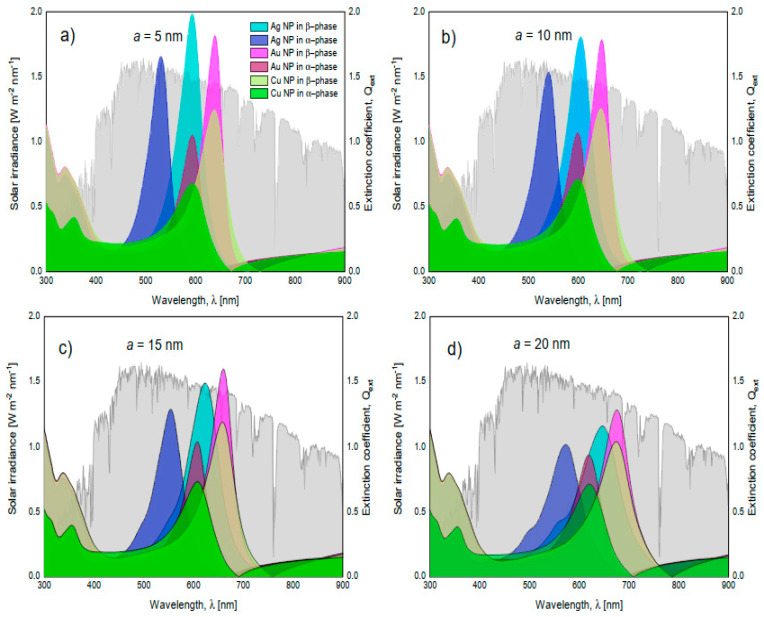
Superposition of the solar spectral irradiance at an air mass of 1.5 (in gray and associated to the left axis) and the extinction coefficients of MoO_3_ films in its alpha and beta phase (right axis). (**a**) nanoparticles with a radius of 5 nm, (**b**) nanoparticles with a radius of 10 nm, (**c**) nanoparticles with a radius of 15 nm, and (**d**) nanoparticles with a radius of 20 nm. In all these cases the *f* = 5%.

Finally, to complete the discussion of the effects of nanoparticle size and nanoparticle type on extinction spectra, Figure 10a,d is presented. In this figure, the solar spectral irradiance at an air mass of 1.5 is superimposed with the different extinction spectra by varying the phase of MoO_3_, NP type, and nanoparticle size. The volume filling fraction was set at 5%; which, as previously discussed, is where the signal is the most intense. The first thing to discuss is the phase change of MoO_3_. In all comparative cases, it is observed that in the *β*-phase of MoO_3_, the extinction coefficient peaks display the highest intensity when compared to the *α*-phase. This behavior is important to highlight due to the advantages in technological applications where this information could be used to simulate the visible radiation trapping layer by modifying only the molybdenum oxide phase and corroborates what was found previously [22]. Additionally, by increasing the radius size of the nanoparticle from 5 to 20 nm, the SPR signal shows a redshift in all comparative cases. This is a feature of resonant plasmonic nanoparticles [48,49]. This behavior is due to a strong interaction between the nanoparticles, which is explained by the increase in the concentration of nanoparticles and the increase in the radius of the nanoparticles and corroborated by experimental works [50,51,52]. Therefore, this work supports what was found in those experimental works, adding a theoretical technique, which is the effective medium theory, to characterize these kinds of doped films. However, it is important to highlight that doping the MoO_3_ film with silver nanoparticles with the smallest radius (Figure 10a), presents a higher extinction coefficient than the Au and Cu nanoparticles. Something to note is that the maximum intensity can be modulated to different wavelengths. On one hand, in Figure 10b this behavior is altered by increasing the size of the nanoparticles, and the MoO_3_ film which presents a slightly higher response in the extinction coefficient is for the MoO_3_ film doped with Ag, but these differences are not significant. On the other hand, modifying the nanoparticle radius to 15 nm causes a significant change in the response of the extinction spectrum, see Figure 10c. The MoO_3_ film with the maximum extinction peak is the Au doped one. Therefore, modifying the nanoparticle radii size enhanced the response of the extinction coefficient, in amplitude, for the cases of Au-NPs, but for Ag-NPs and Cu-NPs, increasing the size of the NPs the extinction coefficient decreased in intensity, as compared with the silver case (see Figure 10). Finally, Figure 10d, depicts the evolution of the extinction coefficient for a fixed radius of nanoparticles of 20 nm. The Au doped MoO_3_ film still shows a better response than the other NP types. At this point the Cu doped MoO_3_ thin films present a good response in the extinction coefficient nearby to the extinction coefficients of the Ag and Au doped MoO_3_ thin films. In addition to the observation of the redshift, the broadening of the extinction coefficient peak can be seen which has also been found experimentally by Ye et al. and Huang et al. [51,52], but that this parameter is not studied in detail even when the increase in the FWHM value is observed.

## 4. Summary and Conclusions

Developing new kinds of materials in solar energy applications brings new and different expectations. Is usual to combine materials that are already well characterized although it is not easy experimentally to predict these behaviors. So in this way, this paper could be useful. We used an effective medium theory to characterize thin films doped with resonant nanoparticles. The effective medium used is usually called the effective refractive index of van de Hulst which is a simple formula and easy to combine with well-establish theory, despite this theory being intended describe colloidal media and not having been adapted for the description of thin films. In this case, we used the optical reflectance and transmittance to study the doped films in a different way. As far as we know, there are few studies related to the extinction coefficient. We focused on the absorber layer. Study of the extinction coefficient is an essential parameter since if the material shows a bigger extinction coefficient, it shows that this layer absorbs more light. As it was presented throughout the manuscript, the changes in the spectra, which can be visualized in the absorption coefficient, are not strong but studying the extinction coefficient, these features are more evident, at least in the intensity of the resonance peaks as the radius of the nanoparticles increases. With this in mind we proposed to use the effective refractive index combined with reflection and transmission coefficients to describe the extinction coefficient as the technique to characterize MoO_3_ thin films doped with resonant nanoparticles for solar energy applications. It was found from the simulations, that the type of nanoparticle embedded in the *α*- and *β*-phase MoO_3_ thin films, the change of the nanoparticle radii and the volume filling fraction play very important roles in enhancing the solar radiation absorbance. Particularly, the copper nanoparticles present similar behavior to the gold NPs in the *β*-phase, which suggests an excellent option in technological applications since using of copper instead of gold could be a cheaper solution. Also, the size of the NPs was shown to be very important combined with the volume filling fraction. Therefore, these two parameters can be chosen depending on the application. Finally, it can be concluded that the phase of the MoO_3_ were the NPs are immersed is very important and gives different results. The extinction coefficients show that the amorphous phase intensifies all the cases compared with crystalline phase of molybdenum trioxide.

## Figures and Tables

**Table 1:** Centered resonant peak values at different phases of MoO_3_ thin films doped with different nanoparticles at different volume filling fractions (*f*).

		Resonant Peak Redshift [nm]									
		*α*-Phase Radii NPs					*β*-Phase Radii NPs				
Nanoparticle	*f* (%)	5	10	15	20	Δ_SPR-*α*_	5	10	15	20	Δ_SPR-*β*_
Ag	1	535	545	559	580	**45**	597	610	630	653	**56**
	3	532	542	556	576	**44**	594	606	626	650	**56**
	5	531	541	554	574	**43**	593	605	624	647	**54**
Au	1	597	603	611	624	**27**	642	651	664	681	**39**
	3	596	601	609	622	**26**	640	648	661	678	**38**
	5	594	600	608	620	**26**	639	648	660	676	**37**
Cu	1	600	605	613	626	**26**	642	650	663	679	**37**
	3	598	604	612	625	**27**	640	648	661	677	**37**
	5	597	602	610	623	**26**	639	647	659	675	**36**

**Table 2:** Full width at half maximum values of resonant peaks at different phases of MoO_3_ thin films doped with different nanoparticles at different volume filling fractions (*f*).

		Full Width at Half Maximum (FWHM) [nm]									
		*α*-Phase Radii NPs					*β*-Phase Radii NPs				
Nanoparticle	*f* (%)	5	10	15	20	Δ_FWHM-_*_α_*	5	10	15	20	Δ_FWHM-_*_β_*
Ag	1	37.12	40.44	50.8	65.81	**28.69**	43.19	51.23	66.15	88.96	**45.77**
	3	41.94	44.89	56.55	72.14	**30.20**	47.68	54.14	68.52	89.92	**42.24**
	5	46.92	49.44	61.13	77.85	**30.93**	53.52	59.62	72.99	94.15	**40.63**
Au	1	38.92	38.78	42.94	51.51	**12.59**	37.38	38.57	45.26	61.09	**23.71**
	3	42.57	42.65	46.92	56.77	**14.20**	40.33	42.21	48.68	64.09	**23.76**
	5	45.61	45.86	49.89	60.37	**14.76**	43.96	46.26	52.98	67.97	**24.01**
Cu	1	61.62	60.57	62.2	66.81	**5.19**	58.52	59.07	63.83	75.6	**17.08**
	3	64.52	63.99	66.1	71.56	**7.04**	57.57	58.5	63.61	74.49	**16.92**
	5	67.11	66.88	69.15	75.31	**8.20**	60.01	61.16	66.52	77.73	**17.72**
